# A time course analysis of the extracellular proteome of *Aspergillus nidulans* growing on sorghum stover

**DOI:** 10.1186/1754-6834-5-52

**Published:** 2012-07-26

**Authors:** Sayali Saykhedkar, Anamika Ray, Patricia Ayoubi-Canaan, Steven D Hartson, Rolf Prade, Andrew J Mort

**Affiliations:** 1 Department of Biochemistry and Molecular Biology, Oklahoma State University, Stillwater, OK, 74078, USA; 2 Department of Microbiology and Molecular Genetics, Oklahoma State University, Stillwater, OK, 74078, USA

**Keywords:** Cellulose, Biofuels, Lignocellulosic biocoversion, *A. nidulans*, Sorghum, Enzymatic hydrolysis, Proteome

## Abstract

**Background:**

Fungi are important players in the turnover of plant biomass because they produce a broad range of degradative enzymes. *Aspergillus nidulans,* a well-studied saprophyte and close homologue to industrially important species such as *A. niger* and *A. oryzae*, was selected for this study.

**Results:**

*A. nidulans* was grown on sorghum stover under solid-state culture conditions for 1, 2, 3, 5, 7 and 14 days. Based on analysis of chitin content, *A. nidulans* grew to be 4-5% of the total biomass in the culture after 2 days and then maintained a steady state of 4% of the total biomass for the next 12 days. A hyphal mat developed on the surface of the sorghum by day one and as seen by scanning electron microscopy the hyphae enmeshed the sorghum particles by day 5. After 14 days hyphae had penetrated the entire sorghum slurry. Analysis (1-D PAGE LC-MS/MS) of the secretome of *A. nidulans*, and analysis of the breakdown products from the sorghum stover showed a wide range of enzymes secreted. A total of 294 extracellular proteins were identified with hemicellulases, cellulases, polygalacturonases, chitinases, esterases and lipases predominating the secretome. Time course analysis revealed a total of 196, 166, 172 and 182 proteins on day 1, 3, 7 and 14 respectively. The fungus used 20% of the xylan and cellulose by day 7 and 30% by day 14. Cellobiose dehydrogenase, feruloyl esterases, and CAZy family 61 endoglucanases, all of which are thought to reduce the recalcitrance of biomass to hydrolysis, were found in high abundance.

**Conclusions:**

Our results show that *A. nidulans* secretes a wide array of enzymes to degrade the major polysaccharides and lipids (but probably not lignin) by 1 day of growth on sorghum. The data suggests simultaneous breakdown of hemicellulose, cellulose and pectin. Despite secretion of most of the enzymes on day 1, changes in the relative abundances of enzymes over the time course indicates that the set of enzymes secreted is tailored to the specific substrates available. Our findings reveal that A*. nidulans* is capable of degrading the major polysaccharides in sorghum without any chemical pre-treatment.

## Introduction

Lignocellulose, a major structural component of woody and non-woody plants, is abundant in nature and has a potential for bioconversion [1]. Lignocellulosic feedstocks like sorghum stover can contribute to abundant fermentable sugars after enzymatic treatment because 60% of its dry weight is cellulose and hemicelluloses [2]. Further it is drought tolerant, noninvasive, grows robustly, has low water requirement and a commercially viable annual crop producing up to 56 metric tons of dry biomass per hectare in the USA [3].

The major challenge imposed by plant cell walls to enzymatic hydrolysis is recalcitrance due to the complexity of the network of lignin, hemicelluloses and cellulose and the crystalinity of cellulose. Different processes including pretreatment with dilute acids, hot water, ammonia fiber explosion and treatment with FeCl_3_ have been tested to overcome this challenge [4-7]. The drawbacks of these pretreatments are accumulation of inhibitory compounds, use of harsh chemicals, hence the expense of the equipment, and lack of reaction specificity [8,9].

Fungi achieve lignocellulosic conversion under mild conditions, albeit rather slowly. Fungi are known to secrete hydrolytic enzymes that are responsible for polysaccharide degradation, but also must produce ligninolytic systems and enzymes to decrease the crystalinity of cellulose [1,10-12]. Here we present a description of the enzymes secreted by *A. nidulans* to support its growth on powdered sorghum stover. *A. nidulans* is a model saprophytic fungus, with a sequenced and annotated genome [13-15]. It is also a well-known producer of plant cell wall degrading enzymes [16,17]. Several proteomic studies on extracellular proteins from aspergillus species growing on various carbon sources have been reported [11,12,18-20] . However, no studies have been reported on growth of *A. nidulans* on sorghum to elucidate the comprehensive strategy of *A. nidulans* for degradation of plant cell walls.

In this study we grew *A. nidulans* on sorghum stover under solid state culture conditions to simulate the natural environment of the fungus. We aimed to identify all secreted enzymes involved in degradation of the sorghum over a time course of 1, 2, 3, 5, 7 and 14 days and in 1% glucose grown cultures. Results from the study of growth, enzyme activities, quantification of breakdown products from the enzymes, and the nature of the remaining undigested sorghum should enhance our understanding of the plant cell wall degradation process and help us to devise ways to accelerate the process of lignocellulosic bioconversion using in vitro enzyme mixtures.

## Results

**Figure 1:**
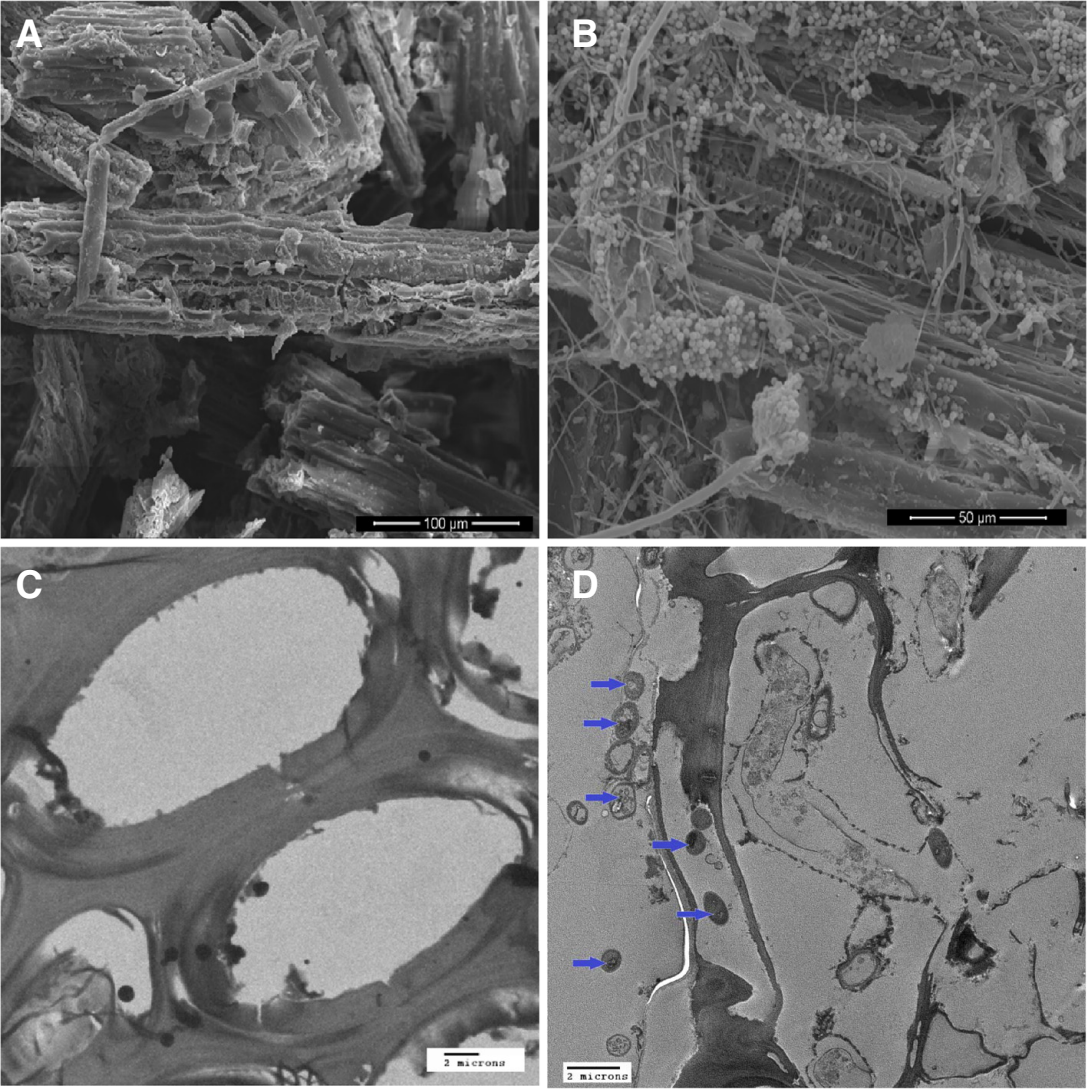
**Growth of*A. nidulans*on sorghum stover. A**. Scanning electron micrograph (SEM) of sorghum particles without any fungal inoculation, **B**. Scanning electron micrograph of sorghum particles enmeshed with fungal mycelia on day 5 showing substantial growth of *A. nidulans*. **C**. Transmission electron micrograph of cross section of sorghum particles without any fungal inoculations. **D**. Cross section of sorghum particles showing presence of fungal particles inside sorghum cells.

To visualize the growth of *A. nidulans* in solid state sorghum cultures, *A. nidulans* samples grown on sorghum stover were sampled on 0, 3 and 5 days after inoculation and analyzed by scanning electron microscopy (SEM) and transmission electron microscopy (TEM). The SEM image in Figure 1A shows uninoculated sorghum particles as a control. Figure 1B indicates dense growth of *A. nidulans* on sorghum stover on day 5 by SEM. Figure 1C is a control depicting only sorghum cell walls imaged by TEM. Figure 1D depicts fungal cells surrounding and within the sorghum cells on day 5 by TEM. By day 1 we saw a mat of fungus covering the surface of the sorghum slurry. The mat appeared to be getting thicker and penetrated throughout the sorghum slurry by day 14.

**Figure 2:**
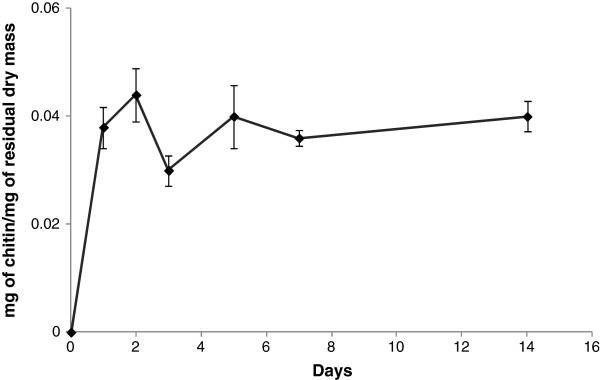
**Growth of fungus on sorghum measured quantitatively by estimation of chitin.** Determination of fungal biomass was done by establishing a conversion factor relating glucosamine to mycelial dry weight. Results are expressed as mg of cells/ mg of dry mass. Dry mass refers to total mass including fungal biomass and residual sorghum. Data represent mean ± SE.

To quantitate the growth of *A. nidulans* in the solid-state cultures, the total chitin content of the cultures was measured. We selected this approach because fungal biomass is difficult to recover and separate from the sorghum particles as the fungal hyphae enmesh and bind tightly to the substrate [21]. Chitin is a long-chain polymer of N-acetyl glucosamine and a key constituent of the fungal cell walls. As no chitin-like materials occur in sorghum stover, determination of chitin content is a good measure of fungal growth. Results from the chitin estimation revealed that *A. nidulans* grew rapidly on day 1 reaching to 3.8% of the total dry biomass on the plate. After day 2 it reached a maximum (4.4% of the total dry biomass) but decreased on day 3 and then became relatively constant for rest of the days (Figure 2).

**Figure 3:**
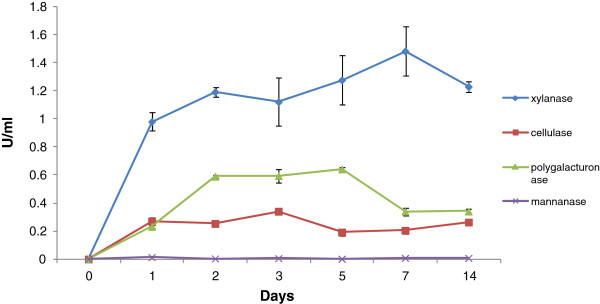
**Estimation of enzyme activities.** Levels of xylanase, cellulase, polygalacturonase and mannanase activities in *A. nidulans* grown on sorghum for 1, 2, 3, 5, 7 and 14 days. Enzyme activities were measured by the released reducing sugars as measured by the 3, 5-dinitrosalicylic acid (DNS) method and expressed as U/ml. One unit of enzyme activity was defined as the amount of enzyme releasing 1 μmol of product per minute. Data represent mean ± SE.

Enzyme activities in the extracellular filtrate (ECF) varied during the growth of *A. nidulans* on the sorghum (Figure 3). Xylanases showed high activity (0.98 U/ml) on day 1 and the activity increased slowly over the next 6 days. Polygalacturonase activity was lower than xylanase activity on days 1 and 2 (0.23 and 0.59 U/ml respectively) with the polygalacturonase activity becoming constant subsequently. Cellulase activity, as measured using carboxymethylcelluose as substrate, reached a fairly constant level of around 0.27 U/ml after day 1. Mannanase activity was not detectable by colorimetric methods. Capillary zone electrophoresis (CZE) analysis, of fluorescently labeled substrates revealed a range of hemicellulase, cellulase, pectinase and mannanase activities in the *A. nidulans* cultures (Data not shown). We did not detect any of the above polysaccharide degrading enzyme activities in ECF collected from glucose control.

The next logical step after determining the enzymatic activities was to identify the individual extracellular enzymes involved in degradation of the sorghum. The *A. nidulans* secretome was analyzed using 1D PAGE LC-MS/MS. Spectrum counts were used to show the relative abundance of the proteins in extracellular filtrates. Spectrum counts are the total number of tandem spectra assigned to each protein and are commonly used to determine relative protein abundances because previous studies have demonstrated a linear correlation between spectrum count and protein abundance in complex samples [22]. The results of the analysis of the secretome are presented in Tables 1,2,3,4,5,6,7,8,9 and 10 and additional file 1: Table S1. A total of 7 proteins were identified in the ECF of 1% glucose grown cultures. These proteins were a hypothetical protein (AN4575), thioredoxin reductase (AN8218), N-acetyl-6-hydroxytryptophan oxidase (AN0231), heat shock protein (AN5129), alkaline protease (AN5558), nucleoside diphosphate kinase (AN8216) and catalase B precursor (AN9339). We identified a total of 196, 166, 172 and 182 proteins on days 1, 3, 7 and 14 respectively during the growth of *A. nidulans* on the sorghum stover. In the sorghum grown samples, eighty-nine proteins were present on all days. Eighty-proteins were exclusively secreted on day 1, whereas only 7, 5 and 19 proteins were unique to days 3, 7 and 14 respectively. Results of LC-MS/MS studies on *A. nidulans* are described below for enzymes directed against different polymers or processes. Where possible the CAZy family affiliation for each enzyme has been noted.

**Table 1:** Identified hemicellulose-degrading proteins and spectrum counts on 1, 3, 7, and 14 days

				**Spectrum count**^**c**^				
**Accession number**^**a**^	**GH family**^**a**^	**Identified proteins**^**a**^	**MW (kDa)**^**b**^	**Day 1**	**Day 3**	**Day 7**	**Day 14**	**SignalP**^**d**^
AN8401	GH3	Beta-1,4-xylosidase	82	60	98	107	132	Y
AN2217	GH3	Beta 1,4-Xylosidase	83	39	48	58	79	Y
AN2359	GH3	Beta-xylosidase	87	53	115	57	0	Y
AN1818	GH10	Beta-1,4-endoxylanase	34	101	142	576	720	Y
AN7401	GH10	Beta-1,4-endoxylanase	38	0	4	11	31	Y
AN3613	GH11	Beta-1,4-endoxylanase A precursor	24	188	174	194	140	Y
AN7152	GH27	Alpha-1,4-galactosidase	69	67	138	124	121	Y
AN8138	GH36	Alpha-1,4-galactosidase	82	0	0	27	24	Y
AN7117	GH39	Xylosidase	50	0	9	13	12	Y
AN8007	GH43	Endoarabinase	34	6	29	20	19	Y
AN2533	GH43	Alpha N-arabinofuranosidase	36	0	13	10	7	Y
AN7781	GH43	Arabinosidase, putative	38	32	74	52	60	Y
AN2534	GH43	Endoarabinase	41	0	13	12	7	Y
AN10919	GH43	1,4-endoxylanase D precursor	42	2	39	50	48	Y
AN7313	GH43	Alpha L-arabinofuranosidase C	52	0	5	0	0	Y
AN7275	GH43	Putative xylosidase	55	0	0	0	24	Y^e^
AN8477	GH43	Xylosidase/arabinofuranosidase	60	37	69	64	97	N^f^
AN5727	GH53	Beta-1,4-endogalactanase	41	11	19	16	18	Y
AN1571	GH54	Alpha-arabinofuranosidase	53	45	98	80	96	Y
AN2632	GH62	Arabinoxylan/arabinofuranohydrolase	33	13	30	29	21	Y
AN7908	GH62	Arabinoxylan/arabinofuranohydrolase	36	27	106	90	113	Y
AN9286	GH67	Alpha-glucuronidase	94	14	17	69	104	Y
AN5061	GH74	Xyloglucanase	88	0	0	0	7	Y
AN2060	GH93	Exo-arabinanase	43	17	24	24	27	Y
AN6093	CE1	Acetyl xylan esterase	34	0	9	6	4	Y
AN1320		Beta-1,4-endoxylanase B	28	10	36	46	55	Y
AN6673		Alpha-fucosidase	92	-	-	30	31	Y
AN9380		Bifunctional xylanase/ deacetylase	26	10	6	9	14	Y

^a^Accession numbers along with protein information and glycosyl hydrolase (GH) family information was obtained from Pedant [23].

^b^Hypothetical molecular weight of the proteins.

^c^Quantifying changes in protein abundance between samples from different time points was done using the spectral count method, yielding a semiquantitative analysis.

^d^SignalP was used to predict secretion signals [23,24].

^e^SignalP as reported at [25].

^f^Not found by SignalP (N-terminal may be incorrectly annotated , a novel signal peptide may be present, or the protein is normally intracellular but was released by autolysis).

**Table 2:** Identified cellulose-degrading proteins and spectrum counts on 1, 3, 7, and 14 days

				**Spectrum count**^**c**^				
**Accession number**^**a**^	**GH family**^**a**^	**Identified proteins**^**a**^	**MW (kDa)**^**b**^	**Day 1**	**Day 3**	**Day 7**	**Day 14**	**SignalP**^**d**^
AN9183	GH1	Beta −1,4-glucosidase	66	11	14	22	14	Y
AN2227	GH3	Beta-1,4-glucosidase	92	9	0	0	0	N^f^
AN2828	GH3	Beta-1,4-glucosidase	78	33	144	131	156	Y
AN4102	GH3	Beta glucosidase	92	78	222	204	215	Y
AN5976	GH3	Beta glucosidase	89	53	105	22	0	Y
AN7396	GH3	Beta glucosidase	84	0	116	107	59	Y
AN1804	GH3	Beta-1,4-glucosidase	68	4	4	49	31	Y
AN10482	GH3	Beta-1,4-glucosidase	94	0	9	21	10	Y
AN1285	GH5	Beta-1,4-endoglucanase	36	21	49	38	42	Y
AN8068	GH5	Putative endoglucanase	63	0	20	46	28	Y
AN9166	GH5	Cellulase family protein	45	0	9	0	5	Y
AN1273	GH6	Cellobiohydrolase	41	12	37	23	39	Y
AN5282	GH6	Cellobiohydrolase	47	0	15	49	54	Y
AN0494	GH7	Cellobiohydrolase	56	15	33	58	80	Y
AN5176	GH7	Cellobiohydrolase	48	63	142	195	234	Y
AN3418	GH7	Beta-1,4-endoglucanase	46	65	82	76	88	Y
AN2664	GH43	Beta-glucanase, putative	55	0	0	0	7	Y
AN3046	GH61	Endoglucanase, putative	32	44	0	0	0	Y
AN3860	GH61	Endoglucanase IV precursor	26	5	0	14	17	Y
AN10419	GH61	Beta-1,4-endoglucanase	29	0	10	10	16	Y
AN6428	GH61	Endoglucanase 4	24	2	0	5	7	Y

^a^Accession numbers along with protein information and glycosyl hydrolase (GH) family information was obtained from Pedant [23].

^b^Hypothetical molecular weight of the proteins.

^c^Quantifying changes in protein abundance between samples from different time points was done using the spectral count method, yielding a semiquantitative analysis.

^d^SignalP was used to predict secretion signals [23,24].

^e^SignalP as reported at [25].

^f^Not found by SignalP (N-terminal may be incorrectly annotated , a novel signal peptide may be present, or the protein is normally intracellular but was released by autolysis).

**Table 3:** Identified pectin-degrading proteins and spectrum counts on 1, 3, 7, and 14 days

				**Spectrum count**^**c**^				
**Accession number**^**a**^	**GH family**^**a**^	**Identified proteins**^**a**^	**MW (kDa)**^**b**^	**Day 1**	**Day 3**	**Day 7**	**Day 14**	**SignalP**^**d**^
AN2463	GH2	Beta-galactosidase	115	0	0	50	96	N^f^
AN2395	GH2	Beta-galactosidase/mannosidase	69	25	70	83	81	Y
AN8761	GH28	Exopolygalaturonase	48	49	38	18	0	Y
AN8891	GH28	Exopolygalaturonase	49	30	20	0	0	Y
AN10274	GH28	Exo-polygalacturonase, putative	46	0	4	0	0	Y
AN0980	GH35	Beta-galactosidase	109	2	14	8	25	Y
AN0756	GH35	Beta-galactosidase	109	0	5	2	8	Y
AN7151	GH78	Alpha-rhamnosidase	100	4	14	64	83	N^f^
AN7828	GH88	Unsaturated rhamnogalacturonan hydrolase	44	11	0	0	0	Y
AN9383	GH105	Unsaturated rhamnogalacturonan hydrolase	43	92	54	60	39	Y
AN0741	PL1	Pectate lyase precursor	35	7	41	28	41	Y
AN2331	PL1	Pectin lyase A precursor	41	17	0	0	0	Y
AN2569	PL1	Pectin lyase A precursor	39	32	29	47	31	Y
AN7646	PL1	Pectate lyase A	35	4	3	19	18	Y
AN6106	PL3	Pectate lyase C	26	6	22	20	23	Y
AN8453	PL3	Pectate lyase C	28	10	0	5	3	Y
AN7135	PL4	Rhamnogalaturonan lyase	56	13	71	71	80	Y
AN4139	PL4	Rhamnogalaturonan lyase	117	6	15	3	5	Y
AN3390	CE8	Pectin methylesterase	35	0	19	11	16	Y
AN4860	CE8	Pectin methylesterase	42	27	3	0	0	Y
AN2528	CE12	Rhamnogalaturonan acetyl esterase	26	4	0	16	16	Y
AN2537		Exopolygalacturonate lyase	44	4	12	6	5	Y

^a^Accession numbers along with protein information and glycosyl hydrolase (GH) family information was obtained from Pedant[23].

^b^Hypothetical molecular weight of the proteins.

^c^Quantifying changes in protein abundance between samples from different time points was done using the spectral count method, yielding a semiquantitative analysis.

^d^SignalP was used to predict secretion signals [23,24].

^e^SignalP as reported at [25].

^f^Not found by SignalP (N-terminal may be incorrectly annotated , a novel signal peptide may be present, or the protein is normally intracellular but was released by autolysis).

**Table 4:** Identified starch degrading proteins and spectrum counts on 1, 3, 7, and 14 days

				**Spectrum count**^**c**^				
**Accession number**^**a**^	**GH family**^**a**^	**Identified proteins**^**a**^	**MW (kDa)**^**b**^	**Day 1**	**Day 3**	**Day 7**	**Day 14**	**SignalP**^**d**^
AN3388	GH13	Alpha amylase	50	33	0	49	41	Y
AN3402	GH13	Alpha amylase	69	11	0	0	0	Y
AN7402	GH15	Glucoamylase	71	7	43	24	15	Y^e^
AN2017	GH31	Alpha-1,4-glucosidase	110	5	12	5	6	Y
AN8953	GH31	Alpha-1,4-glucosidase B	108	85	117	95	121	Y
AN0941	GH31	Alpha-1,4-glucosidase	94	23	24	2	5	Y

^a^Accession numbers along with protein information and glycosyl hydrolase (GH) family information was obtained from Pedant [23].

^b^Hypothetical molecular weight of the proteins.

^c^Quantifying changes in protein abundance between samples from different time points was done using the spectral count method, yielding a semiquantitative analysis.

^d^SignalP was used to predict secretion signals [23,24].

^e^SignalP as reported at [25].

**Table 5:** Identified fungal cell wall degradation/remodeling proteins and spectrum counts on 1, 3, 7, and 14 days

				**Spectrum count**^**c**^				
**Accession number**^**a**^	**GH family**^**a**^	**Identified proteins**^**a**^	**MW (kDa)**^**b**^	**Day 1**	**Day 3**	**Day 7**	**Day 14**	**SignalP**^**d**^
AN0933	GH16	Extracellular cell wall glucanase	42	18	35	11	7	Y
AN0245	GH16	Beta-1,3(4)-endoglucanase, putative	37	0	33	15	29	Y
AN6620	GH16	Beta-1,3(4)-endoglucanase, putative	42	4	0	0	0	Y
AN6819	GH16	Endo-1,3 (4)-glucanase	32	9	7	8	7	Y
AN7950	GH17	Cell wall beta-1,3-endoglucanase	47	17	32	32	26	Y
AN4871	GH18	Protein similar to class V chitinase A	44	5	224	277	317	N^f^
AN8241	GH18	Class III Chi A chitinase	97	0	5	2	0	Y
AN1502	GH20	Protein similar to N-acetylglucosaminidase	68	11	101	124	176	Y
AN0779	GH55	Putative beta-1,3-exoglucanase	84	0	19	19	15	Y
AN4825	GH55	Glucan 1,3-beta glucosidase precursor	97	0	102	108	135	Y
AN9042	GH71	Putative alpha 1,3- glucanase	69	0	51	55	60	Y
AN7657	GH72	1,3-beta-glucanosyltransferase	49	14	37	0	4	Y
AN0472	GH81	Putative beta-1,3-endoglucanase	98	0	102	99	146	Y
AN9339		Catalase B precursor	79	58	111	109	108	Y
AN4390		GPI-anchored cell wall organization protein Ecm33	41	4	7	-	-	Y
AN2385		GPI anchored beta-1,3(4)-endoglucanase, putative	65	3	-	-	-	Y

^a^Accession numbers along with protein information and glycosyl hydrolase (GH) family information was obtained from Pedant [23].

^b^Hypothetical molecular weight of the proteins.

^c^Quantifying changes in protein abundance between samples from different time points was done using the spectral count method, yielding a semiquantitative analysis.

^d^SignalP was used to predict secretion signals [23,24].

^e^SignalP as reported at [25].

^f^Not found by SignalP (N-terminal may be incorrectly annotated , a novel signal peptide may be present, or the protein is normally intracellular but was released by autolysis).

**Table 6:** Identified proteins involved in various plant cell wall modifications and spectrum counts on 1, 3, 7, and 14 days

				**Spectrum count**^**c**^				
**Accession number**^**a**^	**GH family**^**a**^	**Identified proteins**^**a**^	**MW (kDa)**^**b**^	**Day 1**	**Day 3**	**Day 7**	**Day 14**	**SignalP**^**d**^
AN1772		Feruloyl esterase type B	58	105	148	154	142	Y
AN5267	CE1	Feruloyl esterase	28	21	12	56	65	Y
AN5311		Putative tyrosinase	42	14	10	19	19	Y
AN7230		Cellobiose dehydrogenase	83	0	17	39	77	Y

^a^Accession numbers along with protein information and glycosyl hydrolase (GH) family information was obtained from Pedant [23].

^b^Hypothetical molecular weight of the proteins.

^c^Quantifying changes in protein abundance between samples from different time points was done using the spectral count method, yielding a semiquantitative analysis.

^d^SignalP was used to predict secretion signals [23,24].

**Table 7:** Identified mannan degrading proteins and spectrum counts on 1, 3, 7, and 14 days

				**Spectrum count**^**c**^				
**Accession number**^**a**^	**GH family**^**a**^	**Identified proteins**^**a**^	**MW (kDa)**^**b**^	**Day 1**	**Day 3**	**Day 7**	**Day 14**	**SignalP**^**d**^
AN5361	GH2	Beta-1,4-mannosidase	71	0	3	0	0	Y
AN9276	GH5	Beta-1,4-endomannanase	42	0	5	8	15	Y
AN6427	GH5	Beta-1,4-endomannanase	45	0	0	4	6	Y
AN2936	GH38	Alpha-mannosidase	124	0	4	38	72	N^f^
AN0787	GH47	Similar to class I alpha-mannosidase 1B	56	10	48	53	58	Y
AN2325	GH92	Alpha-1,2-mannosidase	82	0	11	15	7	Y
AN1197	GH92	Alpha-1,2-mannosidase	88	0	3	7	6	Y

^a^Accession numbers along with protein information and glycosyl hydrolase (GH) family information was obtained from Pedant [23,24].

^b^Hypothetical molecular weight of the proteins.

^c^Quantifying changes in protein abundance between samples from different time points was done using the spectral count method, yielding a semiquantitative analysis.

^d^SignalP was used to predict secretion signals [23,24].

^e^SignalP as reported at [25].

^f^Not found by SignalP (N-terminal may be incorrectly annotated , a novel signal peptide may be present, or the protein is normally intracellular but was released by autolysis).

**Table 8:** Identified bacterial wall-degrading proteins and spectrum counts on 1, 3, 7, and 14 days

				**Spectrum count**^**c**^				
**Accession number**^**a**^	**GH family**^**a**^	**Identified proteins**^**a**^	**MW (kDa)**^**b**^	**Day 1**	**Day 3**	**Day 7**	**Day 14**	**SignalP**^**d**^
AN6470	GH25	Putative N,O-diacetyl muramidase	23	32	32	23	17	Y
AN8969	GH25	N,O-diacetylmuramidase	23	0	12	0	0	Y

^a^Accession numbers along with protein information and glycosyl hydrolase (GH) family information was obtained from Pedant [23].

^b^Hypothetical molecular weight of the proteins.

^c^Quantifying changes in protein abundance between samples from different time points was done using the spectral count method, yielding a semiquantitative analysis.

^d^SignalP was used to predict secretion signals [23,24].

**Table 9:** Identified proteases and spectrum counts on 1, 3, 7, and 14 days

				**Spectrum count**^**c**^				
**Accession number**^**a**^	**GH family**^**a**^	**Identified proteins**^**a**^	**MW (kDa)**^**b**^	**Day 1**	**Day 3**	**Day 7**	**Day 14**	**SignalP**^**d**^
AN7962		Penicillolysin/deuterolysin metalloprotease	37	170	232	302	318	Y
AN5558		Alkaline protease	42	18	194	65	67	Y
AN2903		Vacuolar aspartyl protease (protienase A)	43	0	41	51	58	Y
AN7159		Hypothetical tripeptidyl-peptidase	71	21	0	0	0	Y
AN10030		Hypothetical serine protease	50	0	12	18	31	Y
AN2366		Hypothetical serine protease	25	11	12	9	6	Y
AN8445		Putative aminopeptidases	54	40	72	74	69	Y
AN1426		Serine carboxypeptidase	62	10	52	51	31	Y
AN7231		Serine carboxypeptidase	57	8	16	9	15	Y
AN0224		Membrane dipeptidase	46	7	9	23	32	Y^e^
AN2572		Dipetidyl dipeptidase	79	9	0	43	91	Y

^a^Accession numbers along with protein information and glycosyl hydrolase (GH) family information was obtained from Pedant [23].

^b^Hypothetical molecular weight of the proteins.

^c^Quantifying changes in protein abundance between samples from different time points was done using the spectral count method, yielding a semiquantitative analysis.

^d^SignalP was used to predict secretion signals [23,24].

^e^SignalP as reported at [25].

**Table 10:** Identified esterases and lipases and spectrum counts on 1, 3, 7, and 14 days

				**Spectrum count**^**c**^				
**Accession number**^**a**^	**GH family**^**a**^	**Identified proteins**^**a**^	**MW (kDa)**^**b**^	**Day 1**	**Day 3**	**Day 7**	**Day 14**	**SignalP**^**d**^
AN5309	CE5	Putative cutinase	22	17	15	39	48	Y
AN7541	CE5	Cutinase, putative	26	23	0	0	0	Y
AN2834	CE12	Esterase, putative	27	6	0	0	0	Y
AN6422	CE16	Cellulose-binding GDSL Lipase/Acylhydrolase	33	0	4	10	9	Y
AN5321		Extracellular lipase putative	62	75	103	71	50	Y
AN9287		GDSL lipase/acylhydrolase	47	23	52	49	50	Y
AN7691		Phosphoesterase superfamily protein	50	23	55	44	13	Y
AN1792		GDSL lipase/acylhydrolase	38	16	54	35	37	Y
AN8046		Putative extracellular lipase	31	11	48	44	47	Y
AN1433		Putative triacylglycerol lipase	60	24	16	6	0	Y
AN1799		Putative lipase	48	0	0	13	39	Y
AN7046		Similar to triacylglycerol lipase	25	3	0	0	0	Y
AN9361		Lipase	63	5	-	-	-	Y

^a^Accession numbers along with protein information and glycosyl hydrolase (GH) family information was obtained from Pedant [23].

^b^Hypothetical molecular weight of the proteins.

^c^Quantifying changes in protein abundance between samples from different time points was done using the spectral count method, yielding a semiquantitative analysis.

^d^SignalP was used to predict secretion signals [23,24].

### Hemicellulose degradation

Hemicellulose is the second most abundant fraction (25-35%) of lignocellulosic biomass [20]. Hydrolysis of hemicelluloses into simple sugars requires multiple enzymes including beta-1,4-endoxylanase, beta-xylosidase, alpha-glucuronidase, alpha-L-arabinofuranosidase and acetyl xylan esterase [26,27]. Beta-endoxylanases cleaves the backbone of xylan and beta-xylosidase releases the xylose units from xylobiose and xylooligomers. Removal of xylan side chains is catalyzed by acetyl xylan esterases, ferulic acid esterases, alpha-L-arabinofuranosidases and alpha-D-glucuronidases [28,29]. Xyloglucanactive beta-1,4-endoglucanase and beta-1,4-glucosidase cleaves xyloglucan backbone and beta-1,4-endomannanase and beta-1,4-mannosidase acts on (galacto-) mannan backbone [28,30]. Twenty-eight different hemicelluloses degrading enzymes were identified in our study (Table 1). A total of twenty *A. nidulans* ORFs have been assigned to GH3 family with three of them being beta-xylosidases [31]. We found all three of these beta-xylosidases (AN8401 AN2359, AN2217). Beta-xylosidases (AN8401 and AN2217) were present in abundance on day 1 and showed a steady increase in spectrum count until day 14. Another beta-xylosidase (AN2359) was present on day 1, 3 and 7 but not on day 14. AN7275, a putative GH43 family xylosidase, only appeared on day 14. We found two of the potential three endoxylanses belonging to GH10 family (AN1818 and AN7401) and one out of the two belonging to GH11 family (AN3613). Beta-1,4-endoxylanase (AN1818) showed a dramatic increase in spectrum count from 101 on day 1 to 720 on day 14. AN3613 started with a high spectrum count on day 1 and then reached a plateau. AN7401 (which has a CBM) was present in lower abundance compared to the other xylanases. An alpha-1,4-galactosidase belonging to family GH27 and another belonging to GH36 (AN7152 and AN8138) were identified. AN7152 was found in high abundance on all 4 days, whereas AN8138 was only secreted on day 7 and 14. Eight proteins (AN2533, AN7781, AN8007, AN2534, AN7275, AN8477, AN10919 and AN7313) belonging to family GH43 were identified. Out of these eight proteins, AN7275 and AN8477 do not have a signal peptide. AN7781 was secreted on all days. Its spectrum count increased more than twice from day 1 to day 3 and then it was constant on day 7 and 14. We identified an arabinosidase from family GH53 (AN1571), and two other arabinosidases (AN7908 and AN2632) belonging to family GH62. We also found one enzyme belonging to family GH53 (endogalactanase), one from GH67 (alpha-glucuronidase), and one from GH93 (exo-arabinase). Most of the hemicellulases were secreted on all days except for nine hemicellulases, which were absent on day 1 but secreted on all other days. One xyloglucanase (AN5061) appeared on day 14 only whereas galactosidase (AN8138) was found on only day 7 and 14.

### Cellulose degradation

The complete hydrolysis of cellulose requires random cleavage of internal bonds by endoglucanases, removal of cellobiose by cellobiohydrolases (exoglucanases), and release of glucose from cellobiose by beta-glucosidase [32-35]. Twenty-one different enzymes likely to be involved in cellulose degradation were identified in our cultures (Table 2). One beta-glucosidase (AN9183) belonging to family GH1 and seven beta-glucosidases belonging to family GH3 were detected. All of the family GH3 enzymes except AN2227 had recognizable signal peptide sequences. Beta-1,4-glucosidase (AN2828 and AN4102) showed high spectrum counts on all days, whereas AN7396 and AN5976 were absent on day 1 and day 14 respectively. Cellobiohydrolases are assigned to family GH6 and GH7. To date only family GH7 cellobiohydrolases have been characterized in *A. nidulans*[36] out of four predicted cellobiohydrolases belonging to family GH6 and GH7 from the *A. nidulans* genome sequence. We identified two cellobiohydrolases (AN1273 and AN5282) from family GH6 and two cellobiohydrolases (AN0494 and AN5176) belonging to family GH7. All showed a gradual increase in spectrum count from day 1 to day 14. Notably cellobiohydrolase (AN5176) showed high spectrum count of 234 on day 14 as compared to other cellobiohydrolases, whereas AN5282 was absent on day 1. We found one beta-1,4-endoglucanase (AN3418), from family GH7 and three beta-endoglucanases belonging to family GH5. Endoglucanases (AN1285 and AN8068) showed relatively low spectrum counts compared to the beta-endoglucanases from family GH7. A cellulase family protein (AN9166) was only present on day 3 and 14. We identified four beta-1,4-endoglucanases from family GH61 (AN10419, AN6428, AN3860 and AN3046) out of nine predicted ORFs for GH61 family proteins in the genome. Out of a total of twenty-one cellulases, seven enzymes were not secreted on day 1 whereas one putative endoglucanase AN2664 appeared only on day 14.

### Pectin degradation

Due to the complex structure of pectin an array of enzymes are needed for its degradation [37,38]. We identified twenty-two enzymes involved in pectin degradation (Table 3). The GH2 family contains beta-galactosidase, beta-mannosidase and beta-glucuronidases. We found two GH2 family members (AN2935 and AN2463). Out of these two GH2 family members, AN2395 showed increased spectrum count across all days. GH28 family consists of endo- and exo-polygalacturonases, rhamnogalacturonan hydrolases and xylogalacturonan hyrolases. Based on the *A. nidulans* genome nine ORFs have been assigned to this family. We identified three exopolygalacturonases (AN8761, AN8891, and AN10274) in the secretome. Out of three GH28 family exopolygalacturonases only AN8761 was secreted with high abundance on all days. So far GH78 family contains alpha-L-rhamnosidase exclusively. Seven *A. nidulans* ORFs have been assigned to this family. We found one alpha-rhamnoside from GH78 (AN7151) and it showed a gradual increased in spectrum count across all the days. We identified two pectate and two pectin lyases from PL1 family and two from PL3 family. Four *A. nidulans* ORFs have been assigned to rhamnogalacturonan lyases from family PL4. We identified two members of PL4. Two unsaturated rhamnogalacturonan hydrolases (AN7828 and AN9383), two pectin methylesterases (AN4860 and AN3390), and one rhamnogalacturonan acetylesterase (AN2528) were also identified. With the exception of members from GH2, GH78, GH105 and PL4, the pectinases were expressed at higher levels only in the early growth stages.

### Starch degradation

Ten *A. nidulans* ORFs have been assigned to family GH31. We identified only three alpha-1,4-glucosidases from family GH31, two alpha-amylases from family GH13 and one amylase from family GH15 (Table 4).

### Fungal cell wall remodeling enzymes

Sixteen enzymes, including chitinases and beta-1,3-endoglucanases involved in fungal cell wall modification/degradation were identified (Table 5). Though chitinases and beta-1,3-endoglucanases were present on day 1, the spectrum count of most of them increased notably on day 3.

### Enzymes involved in various plant cell wall modifications

Table 6 reports four enzymes, two feruloyl esterases, a cellobiose dehydrogenase, and a tyrosinase potentially involved in modification of lignin and other phenolics. The cellobiose dehydrogenase may also be involved in modification of cellulose.

### Mannan and bacterial cell wall degrading enzymes

Table 7 and Table 8 show seven mannan degrading enzymes and two bacterial cell wall degrading enzymes respectively.

### Proteases

Table 9 lists eleven fungal extracellular proteases, the production of which increased over time. They included one metallopreotease (AN7962) one alkaline protease, one aspartyl protease and two hypothetical serine proteases, two serine carboxypeptidases (AN1226, AN2237, AN7121), two putative aminopeptidase, an alkaline protease (AN5558), a putative dipeptidyl aminopeptidase (AN6438), and a membrane peptidase (AN7159). Table 10 show thirteen esterases and lipases secreted by *A. nidulans*. Out of these 12 esterases and lipases, seven were secreted with high abundance throughout the time course, whereas the rest of them were only present on one or two time points.

### Miscellaneous proteins

Total 164 proteins were identified in this category including several hypothetical proteins (Additional file 1: Table S1).

**Figure 4:**
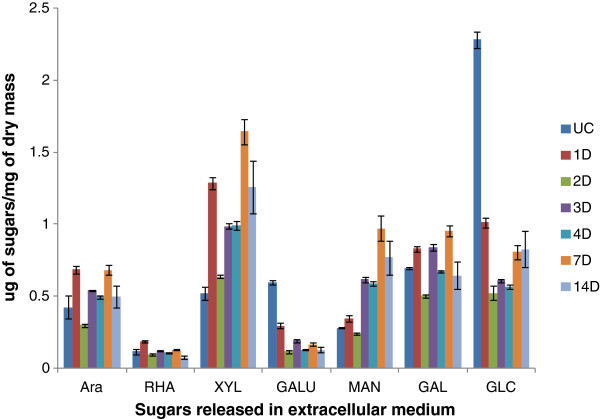
**Estimation of soluble sugars in the extracellular medium of*A. nidulans*grown on sorghum.** This figure depicts breakdown products in the extracellular media after 1, 2, 3, 5, 7 and 14 days. The amounts of each sugar are shown as μg of sugars / mg of dry mass of sorghum. Soluble sugars in the un-inoculated control samples are designated as UC. Data represent mean ± SE.

The presence of high enzyme activities in the extracellular fluid of *A. nidulans* led us to quantify the solubilized breakdown products from the sorghum in the extracellular filtrate (ECF). Soluble polysaccharides and oligosaccharides present in ECF were estimated by gas chromatography after depolymerization by methanolysis and conversion of trimethylsilyl methyl glycosides. Between 0.01% and 0.23% of the total dry mass of the initial sorghum was soluble as each sugar type (Figure 4). The galacturonic acid and glucose content fell dramatically on day 1 and day 2 compared to the control suggesting that these already soluble sugars were readily converted into a form that could be taken up by the fungus. The amounts of arabinose and xylose containing soluble material increased compared to the control, reflecting digestion of the xylan by the enzymes and perhaps a lower ability to take up the solubilized products. The amount of mannose increased fairly consistently from day 1 to day 14. Rhamnose and galactose levels remained relatively constant. The amount of sugars at the different time points was probably a result of the dynamic balance between enzymatic breakdown of sorghum polysaccharides and uptake of sugars by fungus.

**Figure 5:**
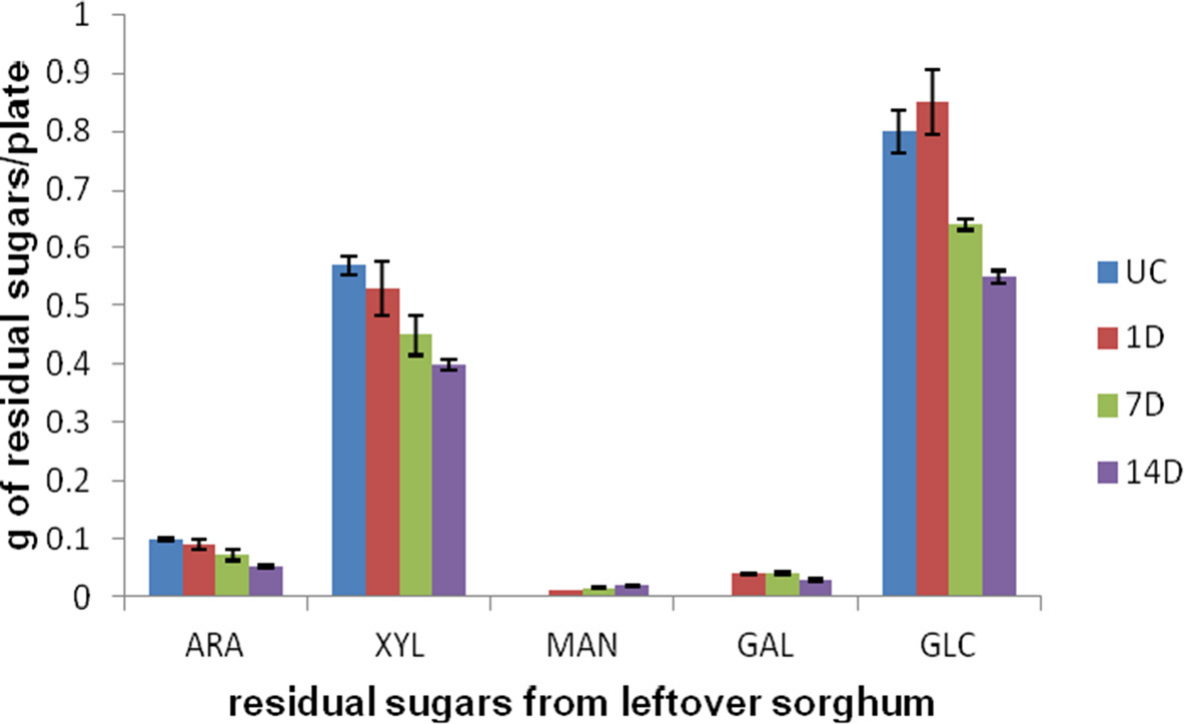
**Estimation of residual sugars of sorghum collected from*A. nidulans*grown on sorghum.** The sugars were estimated using Saeman hydrolysis. The results show the total sugar remaining from the initial 3 g of sorghum after 1, 7 and 14 days of growth of fungus on sorghum. The bars designated as UC show the amount of each sugar in 3 g of un-inoculated sorghum. Data represent mean ± SE.

To find out how effective the fungus was at digesting the sorghum polysaccharides, we quantified the amount of each sugar type remaining on the plate after various times of culture growth (Figure 5). Apart from a slight increase in glucose, which may have come from growth of the fungus or reflect experimental variability, the sorghum sugars decreased steadily. By day 7, 20% of the measurable glucose and xylose had been consumed and by day 14, 30% of these sugars had been utilized. The appearance of mannose and galactose, not present in the uninoculated sorghum, probably reflected the synthesis of fungal galactomannoproteins and mannans.

## Discussion

A major goal of this study was to discover the enzymes *A. nidulans* secretes to digest sorghum stover while growing under conditions mimicking its natural habitat. These findings should help us to design a mixture of enzymes that can convert sorghum stover into readily fermentable sugars in vitro.

From the visual development of a thick fungal mat, the appearance of numerous fungal hyphae in electron microscopic images of the cultures (Figure 1), and the accumulation of chitin during the first two days of culture (Figure 2) we conclude that *A. nidulans* grows vigorously on a semi-solid sorghum slurry. However, although *A. nidulans* maintains a certain level of healthy biomass over a prolonged period, the actual fungal growth seems to reach a plateau after two days. From day 3 onwards we observed high levels of autolytic enzymes in the secretome indicating autolysis of the fungus. Class V chitinase, ChiB, AN4871, belonging to family GH18, N-acetylglucosaminidase, NagA, AN1502, belonging to family GH20, beta-1,3-endoglucanases AN4825 and AN0472, from family GH55 and GH81 respectively, the alkaline protease PrtA, AN5558, metallo-protease PepJ, AN7962, and dipeptidase AN2572 have all been shown to be associated with autolysis [39-42]. All were found to be highly induced from day 3 onwards (Table 5). On the other hand, Catalase B, an enzyme associated with growing and developing hyphae was found in high abundance throughout the time course [43]. Similarly enzymes associated with fungal cell wall synthesis and remodeling such as GPI-anchored cell wall organization protein Ecm33 (AN4390), and putative GPI anchored beta-1,3(4)-endoglucanase (AN2385) [44] were present in the secretome. Based on these findings, we propose that *A. nidulans* is maintaining growth throughout the time course but at the same time is recycling the older hyphae to support growth of hyphal tips and lateral branching. It is well established that lateral branching can be induced by fungal interaction with plants presumably leading to enhanced surface area and nutritional assimilation [45].

Powell et al. [2] studied the chemical composition of conventional grain sorghum, intermediate type sorghum and forage sorghum stover. They reported 31.3% cellulose content in grain type sorghum stover, 25.3% for intermediate sorghum stover, and 29.1% for forage type sorghum on a dry mass basis. The hemicellulose content was found to be 28.2% in grain sorghum stover, 21.7% in intermediate type sorghum stover, and 23.9% in forage type sorghum. The lignin content was between 7 to 7.3% for the three types of sorghum stover. In this study, the cellulose content was found to be 26.6% and the hemicellulose content was 22.3% of the total dry mass (Figure 5).

From the amount of xylanase and cellulase activities we found in the extracellular fluid collected from the culture on day 1 there should have been sufficient enzyme activity to break down all the xylan and cellulose in the sorghum in less than a day. However the concentration of soluble sugars (break-down products from the enzymatic hydrolysis), in the extracellular fluid was low (less than 1 mM), and less than 5% of the total xylan had been digested and seemingly none of the cellulose. This emphasizes the well-known recalcitrance of plant biomass to enzymatic degradation and suggests that the fungus is growing under carbon limited conditions. However, over time, the fungus did appear to be able to digest all classes of cell wall polysaccharides present and it did continue to maintain a certain level of healthy biomass.

We were interested in determining if the fungus attacked the most easily digestible polymers first and then moved on to using the more recalcitrant ones. It has been suggested that fungi first degrade the pectin in plant cell walls to make the hemicelluloses and cellulose more accessible [46]. Sugars characteristic of pectin, i.e. rhamnose and galacturonic acid, were not detected in total hydrolysates of sorghum stover, although both of these sugars were found in the solubilized sugars rinsed from the cultures. This indicates the presence of a small amount of pectin in sorghum. Polygalacturonase activity was induced rapidly in the culture but xylanase and cellulase activity increased at the same time. Looking at the proteomics results one can deduce that almost all of the many enzymes involved in hemicellulose and cellulose degradation are induced in parallel. However in pectin degradation there is a steady decrease over time in exopolygalacturonases, one of the pectin methyl esterases, and one of the pectin lyases. In contrast there was a steep increase in other pectate lyases and rhamnogalacturonan hydrolases.

The recalcitrance of the sorghum or any other plant biomass to digestion is thought to arise from physical inaccessibility of the polymers to the enzymes designed to digest them. Two major factors are the incrustation of the polysaccharides with lignin and the crystalinity of the cellulose. We identified three classes of proteins which might play a key role in overcoming the recalcitrance of the sorghum to digestion.

There is strong evidence that some of the ferulic acids esterified to xylans crosslink to make diferulates which link xylans together thus making them less accessible to enzyme digestion. The ferulates can also be incorporated into lignin thus attaching xylan directly to lignin [47]. Hydrolysis of the ferulate ester bonds is expected to decrease the recalcitrance of biomass to enzymatic hydrolysis [48-50]. Two feruloyl esterases were secreted by *A. nidulans* in high abundance throughout the time course.

Another enzyme in our cultures which might play a role in overcoming recalcitrance of the sorghum to digestion is cellobiose dehydrogenase (CDH). CDHs oxidize cellobiose, or other reducing sugars, and transfer the electrons to Fe^+++^, quiniones, radicals, or oxygen to make hydrogen peroxide [51]. The combination of the resulting Fe^++^ and hydrogen peroxide can lead to the generation of hydroxyl radical which will attack polysaccharides and lignin. At least three putative sequences for CDHs are present in the *A. nidulans* genome. We only detected one form. Interestingly CDH was not present on day 1 and then increased gradually showing a spectrum count of 17 on day 3 up to 77 on day 14. No other studies have reported the secretion of cellobiose dehydrogenases by *A. nidulans* on solid state cultures. The presence of cellobiose dehydrogenase after day 3 may indicate its important role in the degradation of crosslinks between lignin, hemicelluloses and cellulose.

Whenever investigated, GH61 family endoglucanases lack measurable endoglucanase activity, but recently they have been reported to accelerate the hydrolysis of cellulose in cellulase preparations [52,53]. GH61 was initially described as an endoglucanase, but more recently has been shown to be members of the family are copper mono-oxygenases that catalyse cleavage of cellulose oxidatively, releasing cellodextrins [53,54]. Their active site contains a type II copper site, which, after being reduced to Cu^+^ by a reductant such as ascorbate or gallate, is thought to activate oxygen which can then oxidize glycosidic linkages on the surface of crystalline cellulose. This generates new chain ends rendering the substrate far more prone to attack by the classical endoglucanases and cellobiohydrolases [52,53]. Cellobiose dehydrogenase appears to be able to mediate the reduction of the copper site [54]. Recent studies have shown that cellobiose dehydrogenase may enhance cellulose degradation by coupling the oxidation of cellobiose to the reductive activation of copper-dependent polysaccharide monooxygenases [53,54]. Four GH61 enzymes were found in our study.

Studies similar to ours were conducted by Schneider et al. [11] who grew *A. nidulans* and the mesophilic bacteria *Pectobacterium carotovorum* on leaf litter for 20 days, both individually and in co-culture, and identified the proteins secreted into the medium by 1-D PAGE-LC-MS/MS. The aim of their study was to determine the relative contribution of the fungus and the bacterium to the decomposition. They reported a total of 90 proteins secreted by *A. nidulans* on leaf litter during the course of 20 days in comparison with the 294 proteins identified in our sorghum cultures. Seventy- two proteins were found to be common in both sorghum and leaf litter cultures, 59 proteins were exclusively found in the sorghum cultures and 13 were only identified in leaf litter cultures. The identities of the proteins in the two cultures are reported in Additional file 2: Table S2. More than half of the cellulases and xylanases were common to both cultures with two xylanases and four endoglucanases unique to leaf litter cultures. Only one pectinase out of 22 was unique to leaf litter. Interestingly enzymes involved in degradation of crosslinks between lignin, cellulose and hemicelluloses such as cellobiose dehydrogenase and feruloyl esterases were only identified in sorghum cultures. Only half the proteases and less than one third of cell wall remodeling enzymes were common to both the cultures. In another recent study, Couturier et al. [55] identified total 66 proteins in the secretome of *A. nidulans* grown on maize bran. Out of 19 GHs identified in their study in *A. nidulans* secretome, seven hemicellulases belonging to family GH10, GH11, GH39, GH43, GH62 and GH93 and five beta-1,3-glucanases from the GH17, GH55 and GH81 families were identified. They could not detect any beta-1,4-endoglucanase or cellobiohydrolase or any enzyme activity on CMC [55]. The difference in growth and enzyme activities of *A. nidulans* on sorghum and on leaf litter may be attributed to different substrates compositions and growth conditions [12].

## Conclusions

We have identified extracellular proteins secreted during the entire time course of cultivation of *A. nidulans* on sorghum stover. The aim was to learn the cocktail of enzymes we can devise to hydrolyze lignocellulosic biomass efficiently under mild conditions. In this study we identified a total of 294 proteins including cellulases, hemicellulases, pectinases, carbohydrate esterases, chitinases, and many proteins of unknown function. The enzymes such as feruloyl esterases, cellobiose dehydrogenase, and the family GH61 endoglucanases can be important in accelerating biomass conversion by reducing the recalcitrance of cellulose. Some of the hypothetical proteins we found may work as non-hydrolytic accessory proteins aiding hydrolytic enzymatic efficiency. Further work involving global/whole transcriptome studies of *A. nidulans* during its growth on sorghum may give insight into their function. Cloning and expression of the enzymes such as feruloyl esterases, cellobiose dehydrogenase and family 61 endoglucanases will let us test directly if they have beneficial effects.

## Materials and methods

### Growth conditions

*Aspergillus nidulans* Strain A78 was obtained from the fungal genetic stock center (FGSC) [56]. 3 g of ground sorghum (variety mix of AtX2752/RtX2783 and AtX2752/RtX430) was ground in a Thomas Wiley® Mini-Mill (Thomas Scientific, Swedesboro, NJ, USA) by passing through a 60-mesh screen. Fungal spores were counted using a hemacytometer (Spectrum Scientifics, Philadelphia, PA, USA) and adjusted to a million spores per milliliter in water. The ground sorghum was moistened with 6 ml water in a petri plate and autoclaved for an hour at 121^0^C. To these petri plates 15 ml of minimal media (pH 6.5; 95 ml of water, 5 ml 20X nitrate salts and 0.1 ml 1000X trace elements) and 1 ml of spore suspension containing 10^6^ spores was added. Then the petri plates were incubated at 37^0^C and 70% of relative humidity for 1, 2, 3, 5, 7 and 14 days. Uninoculated sorghum stover incubated in same conditions as inoculated stover was used as control. All the chemicals used in this study were purchased from Sigma-Aldrich (St. Louis, MO, USA), unless otherwise stated. Fungus grown on borosilicate 3 mm solid glass beads (Aldrich, Milwaukee, WI) in 1% glucose for 34 hours was used as a control for comparison of secretomes. *A. nidulans* cultured in 1% glucose for 34 hrs utilized only 1/4^th^ of the initial glucose and thus should not be undergoing starvation.

### Sample preparations for microscopy

For scanning electron microscopy (SEM), fungal samples approximately, 5 mm squares, were cut from the plates and placed in the first fixative solution (2.5% glutaraldehyde in 0.2 M sodium phosphate, pH 7.2) and incubated for 2 h. The glutaraldehyde solution was removed and replaced with 0.2 M sodium phosphate buffer, pH 7.2 and samples were washed 3X for 20 min each. Post fixation was done in 1% osmium tetraoxide in dH_2_O for an hour. Dehydration was done with a series of ethanol solutions (50, 70, 90, 95,100,100,100%), for 15 minutes each. Samples were critical point dried and mounted on aluminum stubs using silver paint adhesive followed by coating with gold/palladium. Samples were observed using an FEI quanta 600 electron microscopy (FEI, Hillsboro, OR, USA).

For transmission electron microscopy (TEM), the fixation was done as for the SEM samples. Post fixation was done in 1% osmium tetroxide /1.5% potassium ferricyanide in 0.1% sodium cacodylate buffer for 1 hour. Using a series of ethanol solutions (as described in SEM protocol), samples were dehydrated for 15 minutes each. The dehydrated samples were washed 2X with 100% propylene oxide and then incubation overnight in propylene oxide/polyresin/bed 812 overnight (Polysciences Inc., Warrington, PA, USA) followed by immersion in polyresin/ bed 812 and polymerized at 60^0^C. Samples were sectioned using a Leica EMCU 6 ultramicrotome (Midwest Lab Equipment, Iowa City, IA, USA). Sectioned samples were placed on nickel; carbon/formavar coated grids and stained with uranyl acetate and lead citrate and observed using a JEOL 2100 Transmission electron microscope (JEOL, Austin, TX, USA).

### Estimation of fungal growth by chitin estimation

The entire plate contents of *A. nidulans* cultures after day 1, 2, 3, 5, and 14 was freeze-dried and then mixed thoroughly. One mg samples were transferred to vials with teflon lined caps and hydrolyzed in 3 ml of 6 N HCl, at 100^0^ C for 6 h. The HCl was evaporated overnight in a speed-vac [57]. Once the extracts were dried, 10 μl of 1 M ammonium hydroxide was added to neutralize any residual HCl. Then 10 μl of 100 nanomoles methyl glucamine was added as an internal standard, followed by 100 μl of 20 mg/ml potassium borohydride in DMSO and incubated at 40^0^C for 90 min to reduce hexosamines to hexosaminitols. After 90 min the reaction was stopped by adding 10 μl glacial acetic acid, followed by addition of 20 μl methyl imidazole and 200 μl of acetic anhydride and incubated for 10 min at room temperature to acetylate the hexosaminitols [58,59]. The reaction was stopped by adding 500 μl water and the alditol acetates were purified by adsorbing them to a C18 sep-pak and desorbing them with methylene chloride to remove the polyphoenols. The dichloromethane was then evaporated to dryness. 25 μl of ethyl acetate was added to the dried sample, out of which, 1 μl was injected into the gas chromatograph (Agilent, Santa Clara, CA, USA). A standard curve was prepared using known amounts of glucosamine ranging from 10 to 250 nmol. The glucosamine content of 1 mg aliquots of dried *A. nidulans* grown in liquid media (49.50 μg) was used as a conversion factor to estimate milligrams of fungus per milligram of dry mass.

### Estimation of enzyme activities by capillary zone electrophoresis (CZE) and dinitrosalicylic acid (DNS) assay

Extracellular extracts were collected by washing the whole petri plate contents with 5 ml of autoclaved water and filtering the extract using Whatman no 1 filter paper. Enzyme activities were detected semi-quantitatively by CZE using 8-aminopyrene-1,3,6-trisulfonate (APTS) labeled substrates (xylohexose, cellopentose, arabinoheptaose, mannohexose) [60]. 1 μl aliquots of extracellular extract and 2 μl of respective APTS labeled substrates were added to 22 μl of 50 mM ammonium acetate buffer of optimum pH for the respective enzymes to make a total volume of 25 μl. Samples were incubated for 1 h at 37^0^C and the progress of enzymes was followed by CZE (Bio-Rad, Hercules, CA, USA).

The DNS assay, developed by Sumner and Graham [61] for determination of reducing sugar, was used for quantitative determination of enzyme activity. We used a DNS reagent composed of 0.75% di-nitrosalicylic acid, 0.5% phenol, 0.5% sodium metabisulfite, 1.4% sodium hydroxide, and 21% sodium potassium tartrate [62,63]. To 10 μl of aliquot of extracellular extract, 20 μl of 50 mM ammonium buffer of optimum pH and 20 μl of appropriate substrate (1%W/V) prepared in water, were added. The reaction was carried out in a 96 well PCR plate (Corning, NY, USA) and allowed to incubate for 30 minutes at 37^0^C. The reaction was terminated by addition of 40 μl of DNS mixture to each well and the plate was heated at 100^0^C for 5 min. We read the plate in a 96 well plate reader at 550 nanomoles (Tecan, San Jose, CA, USA). After subtracting substrate blanks from the reading enzyme activities were calculate from standard graphs.

### Estimation of solublized sugars in extracellular filtrate

To identify and quantitate the breakdown products of the polysaccharides, oligosaccharides present in extracellular filtrate were estimated by gas chromatographic (GC) analysis of trimethylsilyl methyl glycosides. Methanolysis and derivatization were performed using the protocol of Chaplin [64] modified by Komalavilas and Mort [65]. A 25 μl aliquot of sample and 100 nanomoles of inositol as an internal standard were dried in 1 dram glass vials in speed vac. 200 μl of methanol 1.5 M of HCI was added to each vial followed by addition of 100 μl of methyl acetate and kept in a heating block at 80^0^C for minimum 3 hours. The vials were cooled, followed by addition of few drops of t-butanol and then evaporated under a stream of nitrogen at room temperatures. Butanol co-evaporates with the HCl, helping to remove the HCl without degrading sugars. 25 μl of 1:1:5 mixture of hexamethyldisilazane: trimethylchlrosilane: pyridine (TMS) was added to all samples. TMS in all the samples was evaporated using nitrogen gas. Dried samples were re-dissolved in 300 μl of isooctane. 1 μl of sample was injected in the gas chromatograph (Agilent, Santa Clara, CA, USA). Standards were made by taking aliquot of 100 nanomoles of each sugar standards and 100 nanomoles of inositol. From the integrator we calculated relative peak areas and used these to calculate how much of each sugar in sample. Area of sugar peak in sample/area of inositol peak in the sample/area of sugar peak in the standards/area of inositol in standards X 100 = number of nanomoles in the sample.

### Liquid chromatography-tandem mass spectroscopy

The fungal cultures grown on sorghum for day 1, 3, 7 and 14 and fungal culture grown in 1% glucose were washed with 5 ml autoclaved water and the filtrates were collected. Concentration of total protein in these extracts was measured by Bradford assay. Small aliquots of the extracellular filtrates containing 50 ug of total protein were run on SDS-PAGE. Gel bands were excised, reduced with Tris (2-carboxyethyl) phosphine), alkylated with 2-Iodoacetamide, and digested for 16 hrs with 8 μg/ml trypsin using ammonium bicarbonate buffer. Peptides were extracted and analyzed by LC-MS/MS using an LTQ-Orbitrap XL hybrid mass spectrometer (Thermo Fisher Scientific, San Jose, CA, USA). For this analysis, an Eksigent spilt less LC pump (Eksigent, Dublin, CA, USA) was used to separate peptide populations on analytical C18 nano columns (Bruker-Michrom Inc., Auburn, CA, USA), with the column effluent being sprayed directly into a new objective pico view ion source. Using a “Big Three” MS/MS method, the Orbitrap analyzer collected an ultra-accurate (R = 60,000) scan of intact peptides, whilst the LTQ ion trap simultaneously performed MS/MS fragmentation analysis of the each of three most abundant peptides eluting in that chromatographic fractions. The LC-MS/MS raw files were used for database searching via the software application Mascot (v2.2.2 from Matrix Science, Boston, MA, USA) against sequences from *A. nidulans* as well as against a sorghum database [23,66] . Searches were validated using Scaffold (Proteome Software Inc., Portland, OR, USA) and the Peptide Prophet algorithm [67]. Criteria for accepting protein identification, was only protein probability thresholds greater than 99% were accepted and at least two peptides needed to be identified, each with 95% certainty. Protein candidates containing similar peptides were grouped to fulfill the principles of parsimony. Search results were also checked for false discoveries using reversed decoy sequence databases, and no decoy sequences were detected, thus bringing our false discovery rate to zero. Changes in protein expression between samples from different time points were examined using the spectral count method [68]. The complete peptide reports from the Scaffold results for samples taken from days 1, 3, 7, and 14 are given in Additional file 3: Table S3, Additional file 4: Table S4, Additional file 5: Table S5, and Additional file 6: Table S6 respectively. SignalP was used to predict secretion signals in the identified proteins [69,70]. Additional information for the presence of a signal peptide was obtained by accessing the following URL [25].

### Estimation of residual sorghum hydrolysates by saeman hydrolysis

Saeman hydrolysis was done on the total biomass on the plates after 1, 7, and 14 days of fungal growth to estimate total sugars [30]. Three hundred mg samples of the thoroughly mixed freeze dried biomass from each petri plate were suspended in 3 ml of 72% sulfuric acid. The samples were incubated at 30^0^ C for 60 min with stirring every 5–10 min followed by dilution of sulfuric acid to 4% by adding 84 ml of water and autoclaved for an hour at 121^0^C. 2 ml aliquot of each sample was neutralized with calcium carbonate to pH 5–6. 25 μl of sample was processed for methanolysis, trimethylsilylation, and gas liquid chromatography (GLC) analysis as described above.

## Abbreviations

1D-PAGE, One dimensional polyacrylamide electrophoresis; LC-MS/MS, Liquid chromatography-tandem mass spectroscopy; CAzY families, Carbohydrate active enzyme families; SEM, Scanning electron microscopy; TEM, Transmission electron microscopy; CZE, Capillary zone electrophoresis; ORF, Open reading frame; GH, Glycosyl hydrolase; AN, Acession number; CBM, Carbohydrate binding module; PL, Pectin lyase; ECF, Extracellular filtrate; CDH, Cellobiose dehydrogenase; CMC, Carboxymethyl cellulose; FGSC, Fungal genetic stock center; APTS, 8-aminopyrene-1,3,6-trisulfonate; DNS, Dinitrosalicylic acid; PCR, Polymer chain reaction; GC, Gas chromatography; TMS, Hexamethyldisilazane: trimethylchlrosilane: pyridine.

## Competing interest

The authors declare that they have no competing interest.

## Authors’ contribution

SS, AR, PAC, SDH, RP, and AJM conceived and designed various aspects of the experiments. SS and AR performed the experiments. SS, AR, PAC, SDH, RP, and AJM al contributed to the analysis of the data, and the writing of the manuscript. All authors read and approved the final manuscript.

## Supplementary Material

Additional file 1 **Table s1.** Identified miscellaneous proteins and spectrum counts on 1, 3, 7, and 14 days. Click here for file

Additional file 2 **Table S2.** Comparison of identified proteins in *A. nidulans* cultures from different carbon sources. Click here for file

Additional file 3 **Table S3.** Protein report of *A. nidulans* grown on sorghum as carbon source on day1. Click here for file

Additional file 4 **Table S4.** Protein report of *A. nidulans* grown on sorghum as carbon source on day3. Click here for file

Additional file 5 **Table S5.** Protein report of *A. nidulans* grown on sorghum as carbon source on day7. Click here for file

Additional file 6 **Table S6.** Protein report of *A. nidulans* grown on sorghum as carbon source on day14. (XLS 1593 kb) Click here for file
